# Sex Differences in Mortality and Receipt of Kidney Replacement Therapy Among Adults With Stage 5 Chronic Kidney Disease

**DOI:** 10.1001/jamainternmed.2025.5979

**Published:** 2025-11-17

**Authors:** Christian Chan, Simon Sawhney, Sofia B. Ahmed, Sandra M. Dumanski, Robert R. Quinn, Ngan N. Lam, Matthew T. James, Pietro Ravani, Ping Liu

**Affiliations:** 1 Department of Medicine, Cumming School of Medicine, University of Calgary, Calgary, Alberta, Canada; 2 Aberdeen Centre for Health Data Science, University of Aberdeen, Aberdeen, Scotland; 3 Faculty of Medicine and Dentistry, University of Alberta, Edmonton, Alberta, Canada; 4 Department of Community Health Sciences, Cumming School of Medicine, University of Calgary, Calgary, Alberta, Canada

## Abstract

**Importance:**

Female individuals typically outlive male individuals in the general population. Whether this survival advantage persists among adults with stage 5 chronic kidney disease (CKD) is unknown.

**Objective:**

To examine sex differences in mortality and treatment with kidney replacement therapy (KRT; including dialysis and transplant) among adults with incident stage 5 CKD.

**Design, Setting, and Participants:**

This population-based cohort study used linked administrative and kidney care program data from Alberta, Canada. Adults 18 years and older with incident non–KRT-dependent stage 5 CKD were identified between April 2005 and March 2019 and observed from study entry until death, out-migration, or March 2021. Data were analyzed from January to August 2025.

**Main Outcomes and Measures:**

Sex-specific, age-stratified standardized mortality ratios were calculated using general population mortality data. Five-year probabilities of all-cause death, receipt of maintenance dialysis, and kidney transplant were estimated in incident stage 5 CKD cases using multistate models, stratified by age and presence of diabetes or cardiovascular disease.

**Results:**

Among 7506 cohort members, 4121 (54.9%) were male (median [IQR] age, 70 [58-80] years), and 3385 (45.1%) were female (median [IQR] age, 74 [61-83] years). The median (IQR) follow-up was 7.9 (4.7-11.5) years. Compared with the general population, female individuals experienced greater excess mortality than male individuals, particularly at younger ages (eg, among adults younger than 55 years: standardized mortality ratio, 40.9 [95% CI, 34.6-47.3] in female individuals vs 15.9 [95% CI, 13.5-18.2] in male individuals); this difference narrowed with increasing age. Within the stage 5 CKD cohort, 5-year all-cause mortality risks were higher in younger female individuals than male individuals (younger than 55 years: 20.7% vs 14.6%) and similar between sexes at older ages. Irrespective of comorbidity, female individuals younger than 65 years were substantially less likely to receive a kidney transplant, and female individuals 65 years and older were less likely to receive dialysis or a transplant.

**Conclusions and Relevance:**

In this cohort study using data from a universal health care system, across all ages, the female survival advantage observed in the general population was absent or even reversed after developing stage 5 CKD. Fewer female individuals transitioned to KRT treatment, independent of their comorbidities. Younger female individuals experienced the greatest survival disadvantage and were less likely to receive a transplant. These findings suggest that differences in treatment decision-making or inequities in access to KRT may contribute to poorer outcomes in female individuals, especially at younger ages. Further investigations are warranted to understand the potential underlying structural, social, and biological mechanisms.

## Key Points

**Question:**

Does the female survival advantage observed in the general population persist among adults with stage 5 chronic kidney disease (CKD)?

**Findings:**

In this cohort study of 7506 adults with incident stage 5 CKD in Alberta, Canada, female individuals were older at disease onset than male individuals. In analyses stratified by age and comorbidities, female individuals younger than 55 years were less likely to survive or receive a kidney transplant than male individuals; female individuals older than 65 years had similar survival but were less likely to receive dialysis.

**Meaning:**

Female individuals may no longer outlive male individuals after developing stage 5 CKD, and their worse outcomes at younger ages warrant further investigation.

## Teaser

This cohort study examines sex differences in mortality and treatment with kidney replacement therapy (including dialysis and transplant) among adults with incident stage 5 chronic kidney disease.

## Introduction

In the general population, female individuals typically live 5 years longer than male individuals, and in nearly all countries, the female survival advantage persists beyond age 65 years.^1^ Whether this survival advantage exists in individuals with advanced chronic diseases, such as stage 5 chronic kidney disease (CKD) or kidney failure, is unknown. Treatment choices for advanced CKD, including dialysis, kidney transplant, or conservative management without kidney replacement therapy (KRT), are complex, potentially influenced by biological, social, and structural factors, and have major implications for health outcomes.

To our knowledge, no population-based study has examined sex differences in mortality and treatment among individuals with incident stage 5 CKD. Prior studies have reported lower hazards of all-cause mortality and death without receiving KRT in female individuals with CKD compared with male individuals.^2,3,4^ However, these studies were based on nephrology-referred cohorts with less advanced disease, limiting generalizability to individuals with stage 5 CKD and potentially introducing bias if referral patterns differ by sex. A study from Australia and New Zealand found that among individuals initiating KRT, female individuals had greater excess mortality than male individuals, particularly among younger patients; kidney transplant reduced but did not eliminate this disparity, with female individuals still experiencing more years of life lost.^5^ However, by conditioning on KRT initiation, that study does not provide insights into those with stage 5 CKD who die before requiring KRT or who choose conservative management. This is a key limitation, as sex and gender may influence CKD progression, treatment decisions, and access to KRT.^6^ Importantly, KRT is not a single event but a longitudinal treatment course that spans the trajectory of stage 5 CKD. Individuals with stage 5 CKD may initiate dialysis, subsequently undergo transplant, or opt for conservative care without dialysis, and sex differences may exist at each transition.

In this population-based study of adults with newly documented stage 5 CKD, we used multistate models to estimate sex-specific probabilities of being in a disease state (stage 5 CKD, dialysis, kidney transplant, and death) at a certain time and the median time spent in each state. Multistate models capture dynamic trajectories by incorporating multiple transition hazards across states.^7,8^ Analyses accounted for mortality rates in the general population and were stratified by age and comorbidity to assess potential sex differences in outcomes. We hypothesized that sex-based differences in disease progression and treatment with KRT may contribute to survival disparities.

## Methods

### Study Design and Data Sources

We conducted a retrospective population-based cohort study (eFigure 1 in Supplement 1) using linked administrative, laboratory, and provincial kidney program data from Alberta, Canada. These databases include demographic characteristics, vital statistics, laboratory results, diagnoses, and procedures for inpatient and ambulatory care,^9^ deterministically linked using a unique Personal Health Number. More than 99% of Alberta residents have universal access to publicly funded hospital and physician services, including serum creatinine testing, dialysis, and kidney transplant. Estimated glomerular filtration rate (eGFR) was calculated using the 2009 Chronic Kidney Disease Epidemiology Collaboration (CKD-EPI) equation without the race coefficient.^10^ Although race data were unavailable, potential measurement bias is likely minimal, as less than 4% of Alberta residents self-identified as Black,^11^ and the 2009 CKD-EPI equation has shown greater accuracy in non-Black populations than the 2021 race-free creatinine equation.^12^ Only outpatient eGFR measurements were used to minimize the inclusion of people with episodes of acute kidney injury. Detailed methods are reported elsewhere.^13^ This study was approved by the Universities of Alberta and Calgary ethics boards, with participant consent waivered due to the retrospective study design using deidentified data. Reporting followed the Reporting of Studies Conducted Using Observational Routinely-Collected Health Data (RECORD) Statement^14^ and nomenclature for kidney function and disease.^15^

### Study Cohort

We included adults 18 years and older with newly documented stage 5 CKD between April 1, 2005, and March 31, 2019. We defined stage 5 CKD following guideline criteria,^16^ requiring a sustained eGFR of less than 15 mL/min/1.73 m^2^ for more than 3 months, with no intermediate values above this threshold. We selected the earliest qualifying period that met the criteria, with the date of the last eGFR in that period (index eGFR) marking study entry. We excluded prevalent cases and individuals who had initiated maintenance KRT at or before study entry, using a minimum 3-year look-back period.

### Exposure

Sex at cohort entry was the study exposure. The Provincial Registry records the legal sex (female or male) annually for all registered Alberta residents since 1994. For most individuals included in this database, legal sex aligns with their sex assigned at birth.^17^ However, transgender and gender-diverse residents have had the option to change their legal sex marker since 2014.^18^ Thus, some individuals’ legal sex at cohort entry might not reflect their sex assigned at birth but rather their current gender identity. To assess this, we examined the number of individuals who had changed their legal sex marker.

### Outcomes

The primary outcome was all-cause death, ascertained from Alberta Vital Statistics.^9^ We measured times from study entry to all-cause death, death without receiving KRT, dialysis initiation, and kidney transplant. The initiation of maintenance dialysis and kidney transplant were identified using provincial kidney program data and administrative records (eTable 1 in Supplement 1). Episodes of dialysis for acute kidney injury or disease were excluded. Follow-up was from study entry until the earliest of death, out-migration, or study end on March 31, 2021.

### Covariates

Baseline characteristics included age, residence, index eGFR, qualifying period, albuminuria, comorbidities, and health care utilization. Age was categorized as 18 to 44 years, 45 to 54 years, 55 to 64 years, 65 to 74 years, 75 to 84 years, and 85 years and older. Urban or rural residence was determined using the Canadian Census and the Statistics Canada Postal Code Conversion File. For the qualifying period, both its duration and the number of outpatient eGFR measurements were obtained. Albuminuria was defined using the most recent outpatient measurement within the previous 3 years, prioritized as urine albumin to creatinine ratio, protein to creatinine ratio, or urine dipstick.^16^ Comorbidities were identified using validated algorithms and included diabetes, cardiovascular disease (defined as at least 1 of the following: congestive heart failure, myocardial infarction, stroke or transient ischemic attack, or peripheral vascular disease), chronic pulmonary disease, cancer, and dementia.^19^ Health care use in the year prior to cohort entry included general practitioner visits, outpatient nephrology consultations, and hospitalizations.

### Statistical Analysis

#### Mortality Rate Estimation

To compare crude mortality rates accounting for background mortality, we estimated sex-specific standardized mortality ratios using annual Alberta data from Statistics Canada (2005 to 2021),^11^ stratified by sex and 5-year age groups, including age 20 years and older owing to data availability. We also calculated female to male mortality rate ratios and 95% CIs for both the patient cohort and the general population.

#### Estimation of Probabilities of Being in Each State Using a Multistate Model

**Figure 1.**
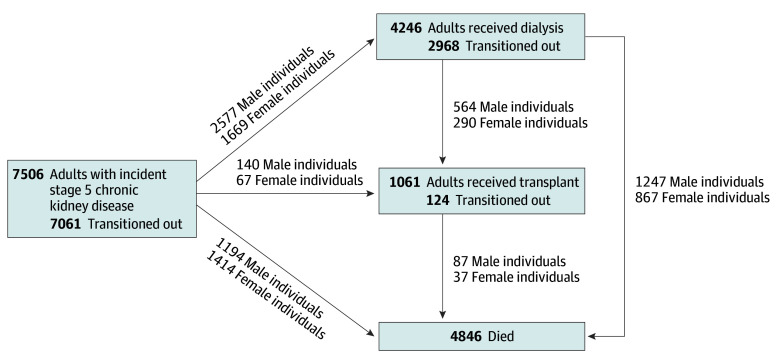
Illustration of the Multistate Model

To examine disease progression and treatment among individuals with stage 5 CKD, we used a 4-state multistate model: stage 5 CKD (initial state), dialysis and kidney transplant (intermediate states), and death (absorbing state). The model allowed 6 transitions: stage 5 CKD to dialysis, transplant, or death; dialysis to transplant or death; and transplant to death (Figure 1). Transitions such as return from dialysis to stage 5 CKD or from transplant to dialysis were not considered due to different care pathways, small numbers, and potential violations of the Markov assumption (future states depend only on the current state, not prior history). Compared with analyzing time to a single event^20^ or competing events,^21^ the multistate model offers 2 advantages. First, it accounts for transitions between intermediate states and from intermediate to absorbing states, such that the probability of being in the dialysis state depends on both the hazard of initiating dialysis and the hazards of exiting via transplant or death. Second, it distinguishes deaths occurring without receiving KRT from those following KRT initiation. We estimated 5-year state occupancy probabilities for death, dialysis, transplant, and stage 5 CKD using the nonparametric Aalen-Johansen estimator via the mstate package in R version 4.5.0 (The R Foundation).^22,23^

#### Other Analyses

We estimated female to male hazard ratios (HRs) using Cox proportional hazards models for all-cause mortality and cause-specific Cox models for transitions from stage 5 CKD to death (ie, death without receiving KRT), dialysis, and transplant, adjusting for baseline characteristics. We estimated the cumulative all-cause mortality risk, median survival, time remaining in the stage 5 CKD state, and, among individuals who started dialysis, time spent in the dialysis state using the Kaplan-Meier method.^20^ IQRs were reported alongside median times. Where applicable, analyses were stratified by sex, age, and comorbidity. Detailed statistical methods are provided in eMethods in Supplement 1. Data were analyzed from January to August 2025.

## Results

### Baseline Characteristics

Among 5.4 million Alberta residents registered from 1994 to 2021 (50.9% male), 7506 individuals with newly documented stage 5 CKD were included in this study. Of these, 4121 (54.9%) were male, and 3385 (45.1%) were female. Five individuals (<0.1%) had changed their legal sex marker before cohort entry (Figure 1; eTable 2 in Supplement 1). Female individuals met stage 5 CKD criteria at older ages than male individuals (median [IQR] age at first documentation of stage 5 CKD, 74 [61-83] years vs 70 [58-80] years), with similar baseline eGFR and qualifying period by sex. Severe albuminuria was less common among female individuals across all ages, while diabetes prevalence was similar. Among older adults, female individuals were less likely to have a history of cancer, chronic pulmonary disease, and outpatient nephrology visits, but they were more likely to have cardiovascular disease, dementia, and prior hospitalizations (Table 1; eTable 3 in Supplement 1).

**Table 1.** Baseline Patient Characteristics

Characteristic	Individuals, No. (%)		
	Total (N = 7506)	Male individuals (n = 4121)	Female individuals (n = 3385)
Age, median (IQR), y	71 (59-81)	70 (58-80)	74 (61-83)
18-44	627 (8.4)	371 (9.0)	256 (7.6)
45-54	763 (10.2)	455 (11.0)	308 (9.1)
55-64	1355 (18.1)	794 (19.3)	561 (16.6)
65-74	1639 (21.8)	968 (23.5)	671 (19.8)
75-84	1916 (25.5)	1005 (24.4)	911 (26.9)
≥85	1206 (16.1)	528 (12.8)	678 (20.0)
Rural location of residence	1317 (17.5)	742 (18.0)	575 (17.0)
Index eGFR, mean (SD), mL/min/1.73 m^2^	11.6 (2.3)	11.5 (2.3)	11.6 (2.4)
Qualifying period, median (IQR)			
Duration, d	107 (97-126)	106 (96-122)	110 (97-131)
Outpatient eGFR measurements, No.	4 (3-6)	4 (3-6)	4 (3-5)
Albuminuria^a^			
A1	681 (9.1)	252 (6.1)	429 (12.7)
A2	1342 (17.9)	647 (15.7)	695 (20.5)
A3	5009 (66.7)	2980 (72.3)	2029 (59.9)
Unmeasured	474 (6.3)	242 (5.9)	232 (6.9)
Comorbidities			
Diabetes and cardiovascular disease			
Neither	1983 (26.4)	1135 (27.5)	848 (25.1)
Diabetes only	1834 (24.4)	1047 (25.4)	787 (23.2)
Cardiovascular disease only	1138 (15.2)	564 (13.7)	574 (17.0)
Both	2551 (34.0)	1375 (33.4)	1176 (34.7)
Chronic pulmonary disease	2194 (29.2)	1191 (28.9)	1003 (29.6)
Cancer	1576 (21.0)	945 (22.9)	631 (18.6)
Dementia	784 (10.4)	323 (7.8)	461 (13.6)
Health care use in the previous year			
General practitioner visit	7266 (96.8)	3951 (95.9)	3315 (97.9)
Outpatient nephrology visit	6041 (80.5)	3480 (84.4)	2561 (75.7)
Hospitalization	3122 (41.6)	1648 (40.0)	1474 (43.5)

Abbreviation: eGFR, estimated glomerular filtration rate.

^a^ Albuminuria severity was categorized as normal to mild (A1), moderate (A2), severe (A3), or unmeasured, corresponding to albumin to creatinine ratio values of less than 30, 30 to 300, or more than 300 mg/g; protein to creatinine ratio values of less than 150, 150 to 500, or more than 500 mg/g; and urine dipstick protein levels of negative or trace values, 1+, or 2+ or more. If multiple albuminuria measurement methods were available, albumin to creatinine ratio was used preferentially, followed by protein to creatinine ratio and urine dipstick.

### Mortality Rates Compared With General Population

**Figure 2.**
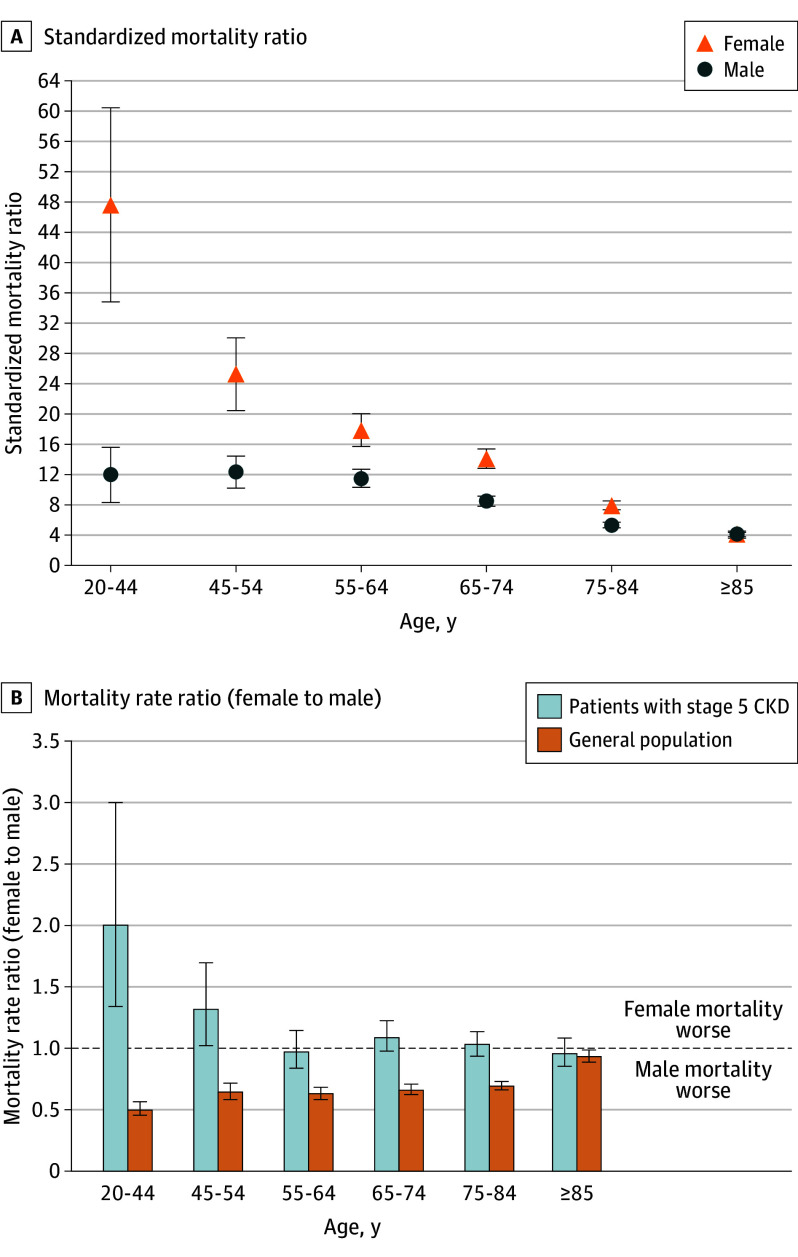
Standardized Mortality Ratio and Female to Male Mortality Rate Ratio CKD indicates chronic kidney disease. Error bars indicate 95% CIs.

Over a median (IQR) follow-up of 7.9 (4.7-11.5) years, female individuals with stage 5 CKD experienced greater excess mortality than male patients, particularly at younger ages. Among those aged 20 to 44 years, standardized mortality ratios were 47.5 (95% CI, 34.7-60.3) for female individuals (31.9 observed deaths vs 0.7 expected deaths per 1000 person-years) and 12.0 (95% CI, 8.3-15.6) for male individuals (16.0 observed deaths vs 1.3 expected deaths per 1000 person-years). This gap narrowed with age: in ages 65 to 74 years, standardized mortality ratios were 14.1 (95% CI, 12.8-15.4) for female individuals (170.0 observed deaths vs 12.1 expected deaths per 1000 person-years) and 8.5 (95% CI, 7.9-9.2) for male individuals (156.0 observed deaths vs 18.3 expected deaths per 1000 person-years), and by age 85 years and older, values were similar (4.2 [95% CI, 3.9-4.5] for female individuals [490.2 observed deaths vs 117.3 expected deaths per 1000 person-years] vs 4.1 [95% CI, 3.7-4.4] for male individuals [510.3 observed deaths vs 125.5 expected deaths per 1000 person-years]). Female to male mortality rate ratios showed consistent patterns: the female survival advantage in the general population was diminished in stage 5 CKD and reversed in younger ages (Figure 2).

### Probabilities of Being in a State

At 5 years from stage 5 CKD diagnosis, 55.1% of individuals had died, 25.8% were receiving dialysis, 11.8% had received a kidney transplant, and 7.3% remained in stage 5 CKD. Female individuals had higher crude risks of all-cause mortality (58.9% [95% CI, 57.2-60.7] vs 51.9% [95% CI, 50.3-53.5]) and death without receiving KRT (39.9% [95% CI, 38.3-41.6] vs 28.3% [95% CI, 26.9-29.7]) but lower probabilities of receiving dialysis (23.3% [95% CI, 21.8-24.8] vs 27.8% [95% CI, 26.3-29.3]) and transplant (8.5% [95% CI, 7.5-9.5] vs 14.5% [95% CI, 13.4-15.7]) than male individuals.

**Figure 3.**
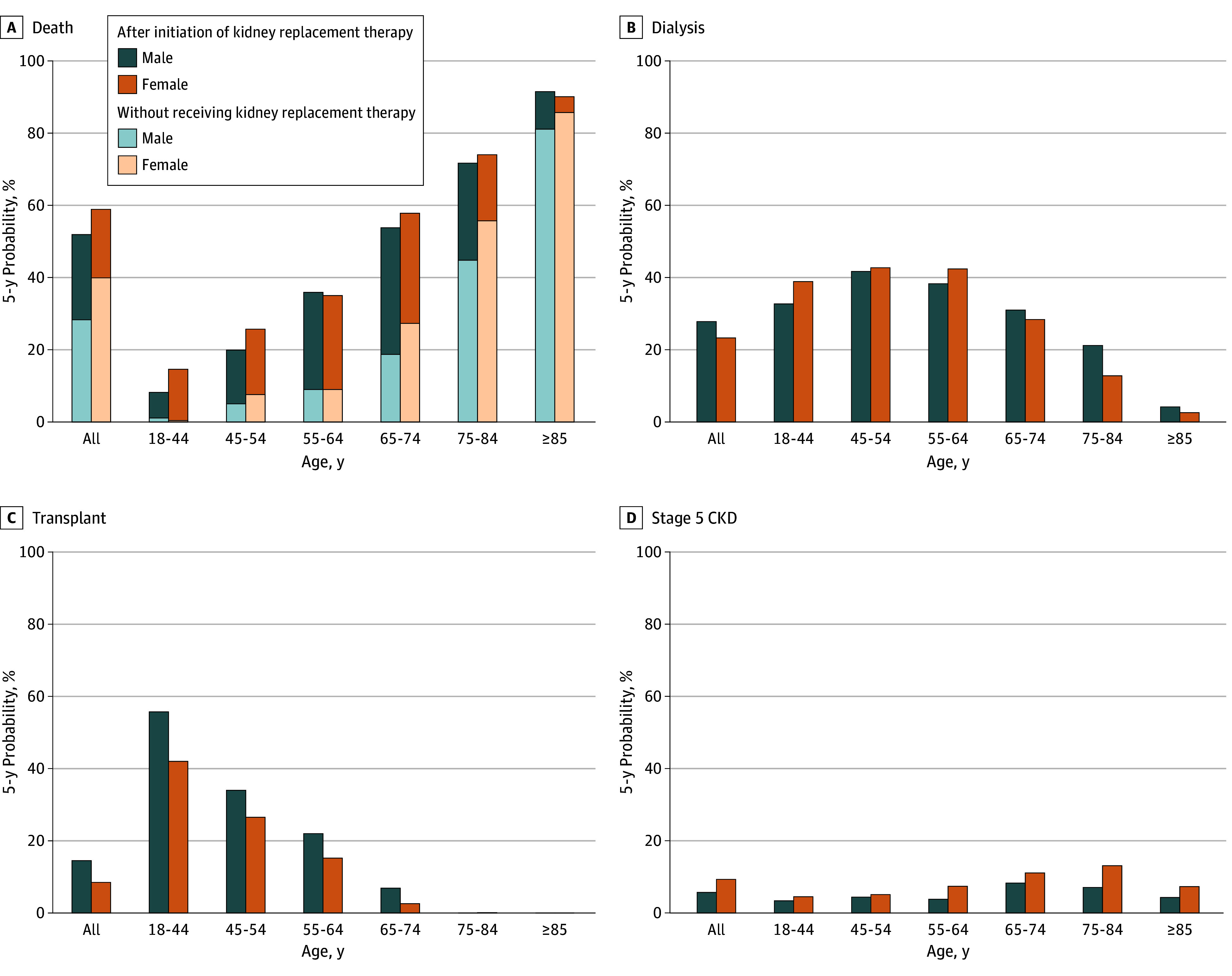
Sex-Specific 5-Year Probabilities of Being in a State, Overall and by Baseline Age Overall risk of death is the sum of the risk of death without receiving kidney replacement therapy and the risk of death following initiation of kidney replacement therapy. CKD indicates chronic kidney disease.

After stratifying by age, 5-year all-cause mortality risks were similar between sexes in older age groups but higher in female individuals younger than 55 years (Figure 3; eFigure 2 in Supplement 1). Compared with male individuals in the same age group, probabilities of receiving dialysis were higher or similar in female individuals younger than 65 years but lower in female individuals 65 years and older. Transplant probabilities declined with age and were consistently lower in female individuals across all age groups. Female individuals had a higher risk of death without receiving KRT except in those younger than 65 years, where patterns were less consistent. Across all ages, female individuals had a higher probability of remaining in stage 5 CKD.

Across comorbidity subgroups defined by diabetes and cardiovascular disease, female individuals had higher risks of all-cause mortality and death without receiving KRT. They had lower probabilities of receiving dialysis and transplant and a higher probability of remaining in stage 5 CKD compared with male individuals. Analyses stratified by both age and comorbidity were generally consistent with age-stratified findings (eTables 4 and 5 in Supplement 1).

### Adjusted HRs of Outcomes

In the overall cohort, sex was not statistically associated with all-cause mortality or death without receiving KRT. However, female individuals had lower adjusted hazards of transitioning from stage 5 CKD to dialysis and to transplant. In age-stratified analyses, female individuals had higher hazards of all-cause mortality in those younger than 45 years, higher hazards of death without receiving KRT among those aged 65 to 74 years, and lower hazards of transitioning to dialysis in those 55 years and older (eTable 6 in Supplement 1).

### Survival Time and Time Spent in Dialysis and Stage 5 CKD States

In crude analyses, male individuals survived longer than female individuals (median [IQR] survival, 4.78 [1.75-11.10] years vs 3.85 [1.39-8.52] years). This trend held across most age and comorbidity subgroups, except among those 85 years and older, where survival was similar (median [IQR] survival, 1.30 [0.44-2.72] years vs 1.31 [0.51-2.70] years). Female individuals spent longer time in the dialysis state if they initiated dialysis (median [IQR] time, 3.69 [1.76-6.31] years vs 3.52 [1.73-5.87] years) and remained longer in stage 5 CKD (median [IQR] time, 1.09 [0.42-2.45] years vs 0.85 [0.35-1.86] years) than male individuals. These trends were consistent across subgroups, except among those aged 75 to 84 years, where female individuals had shorter time in the dialysis state than male individuals (median [IQR] time, 3.00 [1.20-5.19] years vs 3.35 [1.44-5.44] years) (Table 2).

**Table 2.** Median Survival and Median Years Spent in Stage 5 Chronic Kidney Disease (CKD) and Dialysis States (Unstandardized)

Subgroup	Time, median (IQR), y					
	Survival		Stage 5 CKD		Dialysis	
	Male individuals	Female individuals	Male individuals	Female individuals	Male individuals	Female individuals
Overall	4.78 (1.75-11.10)	3.85 (1.39-8.52)	0.85 (0.35-1.86)	1.09 (0.42-2.45)	3.52 (1.73-5.87)	3.69 (1.76-6.31)
Age, y						
18-44	NA (NA-NA)^a^	NA (9.16-NA)^a^	0.53 (0.21-1.22)	0.52 (0.24-1.37)	3.15 (1.53-5.66)	3.76 (1.87-6.54)
45-54	NA (6.47-NA)^a^	12.72 (4.94-NA)^a^	0.65 (0.26-1.34)	0.82 (0.28-1.65)	4.01 (2.16-7.16)	4.19 (2.26-6.99)
55-64	7.51 (3.80-14.36)	7.58 (3.87-13.71)	0.72 (0.32-1.58)	0.95 (0.38-2.11)	3.84 (2.21-6.37)	3.96 (2.20-7.50)
65-74	4.63 (2.08-8.13)	4.26 (1.80-8.04)	0.96 (0.45-2.22)	1.28 (0.48-2.78)	3.38 (1.48-5.49)	3.42 (1.51-6.32)
75-84	2.79 (1.05-5.38)	2.66 (1.11-5.14)	1.04 (0.47-2.14)	1.42 (0.54-3.06)	3.35 (1.44-5.44)	3.00 (1.20-5.19)
≥85	1.30 (0.44-2.72)	1.31 (0.51-2.70)	1.04 (0.33-2.17)	1.10 (0.44-2.28)	2.69 (0.88-3.64)	2.77 (1.45-4.35)
Comorbidities						
Neither diabetes nor CVD	10.56 (3.58-NA)^a^	9.31 (3.26-NA)^a^	0.97 (0.39-2.03)	1.30 (0.52-2.85)	3.59 (1.62-6.08)	4.14 (1.92-7.25)
Diabetes alone	5.93 (2.90-11.16)	5.18 (2.23-10.29)	0.84 (0.36-1.85)	1.07 (0.42-2.62)	3.80 (2.23-6.37)	4.22 (2.17-7.32)
CVD alone	2.61 (0.82-6.32)	2.15 (0.79-4.67)	1.01 (0.40-2.20)	1.19 (0.44-2.69)	3.26 (1.57-5.48)	3.34 (1.31-4.77)
Both diabetes and CVD	3.02 (1.09-6.13)	2.62 (0.99-4.94)	0.72 (0.31-1.57)	0.89 (0.34-2.02)	3.20 (1.46-5.29)	3.20 (1.47-5.54)

Abbreviations: CVD, cardiovascular disease; NA, not applicable.

^a^ The median survival time and lower or upper IQR values were NA because all-cause mortality risk did not exceed 50%, 25%, or 75%, respectively. However, differences were observed in the 10th percentile of survival time; among individuals aged 18 to 44 years, 8.42 years for male individuals and 4.21 years for female individuals; for those aged 45 to 54 years, 2.45 years for male individuals and 2.08 years for female individuals.

## Discussion

In this population-based cohort study within a universal health care system, the typical female survival advantage seen in the general population was absent or reversed in adults with stage 5 CKD, with the greatest disadvantage in younger female individuals. Despite comparable age and comorbidity profiles, female individuals were less likely than male individuals to receive a kidney transplant, even though transplant is generally the preferred treatment for eligible patients. Among patients 65 years and older, overall mortality was similar between sexes; however, female individuals were less likely to receive dialysis, more likely to die without receiving KRT, and more likely to remain in the stage 5 CKD state.

Biological, social, and systemic factors may contribute to the loss or reversal of the typical female survival advantage in stage 5 CKD, particularly among younger patients. In the youngest group (aged 18 to 44 years), higher female mortality was largely driven by deaths following KRT, most often dialysis. This disparity may reflect inadequate dialysis delivery in smaller-bodied individuals (often female individuals) due to limitations in the Kt/V calculation.^21^ It may also reflect worse overall health among female individuals, limiting transplant eligibility, or reduced access to kidney transplant. We observed that female individuals had significantly lower transplant probabilities than male individuals, especially at younger ages, despite similar baseline characteristics. Higher sensitization from pregnancy, especially multiple pregnancies, prolongs wait times and reduces transplant likelihood.^24^ Additional barriers may include inequities in transplant referral and waitlisting,^25,26^ social factors such as caregiving responsibilities and pregnancy planning,^27^ sex-based differences in shared decision-making, and the higher prevalence of autoimmune diseases in younger female individuals, like lupus nephritis, that may complicate transplant eligibility.^28^

Although female individuals met stage 5 CKD criteria later and all-cause mortality was comparable between sexes, differences in disease progression or more conservative treatment among older patients may explain the higher probability of female individuals remaining in stage 5 CKD and dying without receiving KRT. Both outcomes may reflect either nonprogressive disease or preference for conservative, nondialysis management. Slower progression in female individuals may relate to estrogen’s nephroprotective effects, slower eGFR decline, and lower prevalence of severe albuminuria, as reported in our and prior studies.^6,29,30^ Among those aged 65 to 84 years, the absence of a female survival advantage may reflect lower dialysis uptake due to greater reluctance or conservatism during shared decision-making, consistent with our finding that older female individuals were less likely to see a nephrologist before stage 5 CKD. Nevertheless, potential survival advantages from slower disease progression may be offset by systemic inequities. Studies report that female individuals have lower CKD awareness, less frequent monitoring, and fewer nephrology referrals, even within universal health systems.^31,32^ Among those 85 years and older, more conservative choices may reduce treatment burden and hospitalization risk, with female to male mortality ratios resembling the general population. While our dataset lacked information on selection of conservative care, an Australian study found that older adults and female individuals with kidney failure were more likely to choose this modality when referred to nephrologists.^33^ This decision may reflect gender-based preferences or perceptions of dialysis,^34^ the lack of spousal caregiving (as older female individuals are more likely to live alone),^35^ or coexisting conditions. For example, the higher prevalence of dementia among older female individuals in our cohort may discourage health care professionals from offering dialysis.

This study has implications for health policy, clinical care, and research. Identifying and quantifying sex-based disparities in treatment and survival outcomes among individuals with incident stage 5 CKD is a critical first step toward addressing them. By presenting mortality rates accounting for general population mortality and 5-year probabilities of death, receiving dialysis, kidney transplant, and remaining KRT-free, our study provides a more comprehensive view of disease trajectories. These data can inform shared decision-making and guide more equitable resource allocation, including targeted policies for younger disadvantaged groups and supportive care for older patients. To design effective interventions, mixed-methods research should examine biological mechanisms, preventive therapy use, symptom burden, frailty, caregiving support, and structural factors influencing treatment decisions and selective KRT initiation. To reduce disparities in kidney transplant, transplant centers and registries should report referral, waitlisting, and transplant rates by sex and intersecting sociodemographic and clinical factors^24^ and, where feasible, document reasons for exclusion at each stage.

Our study adds to prior studies in less advanced CKD or referred nephrology cohorts, which reported higher hazards of KRT initiation and lower hazards of all-cause mortality or death without receiving KRT in female individuals,^2,3,4^ the study from Australia and New Zealand comparing observed deaths on KRT with expected deaths in the general population, and a US study of KRT decision-making in stage 5 CKD but predominantly included male individuals.^36^ Using population-based data from a geographically defined region with universal health care coverage, we constructed a cohort of adults with incident, non–KRT-dependent stage 5 CKD. We applied internationally recommended criteria to define stage 5 CKD^16^ and used a 3-year look-back period to identify incident cases. A novelty of our study was the comparison of sex differences in stage 5 CKD mortality without conditioning on referral or treatment and accounting for general population mortality. Estimating probabilities of being in different disease and treatment states further provides a more nuanced understanding of how treatment relates to sex-based survival differences.

### Limitations

This study has limitations. First, the findings may not generalize to populations without universal KRT access or with greater ethnic diversity. Many socioeconomic and clinical factors, such as living status, causes of CKD, medication adherence, blood pressure or diabetes control, and smoking status, were not captured in our administrative health databases and may have influenced outcomes and treatment decisions.^6^ Nevertheless, our methodological framework is applicable to other populations. Second, outcome probability analyses stratified by sex, age, and comorbidity had relatively small sample sizes, limiting statistical power. Despite stratification for multiple variables, residual confounding is possible. Finally, the lack of data on treatment preferences and conservative CKD management limits conclusions about the mechanisms underlying these findings.

## Conclusions

In this population-based cohort study, the typical female survival advantage was absent or reversed among adults with stage 5 CKD under universal health care, with the greatest disadvantage in younger female individuals. Inequitable access to transplant may partly explain this finding. Older female individuals were more likely to remain non–KRT-dependent and die without receiving KRT and were less likely to be treated with dialysis, independent of age or major comorbidities. These findings highlight the need for further research into the biological, structural, and societal factors influencing sex differences in treatment decisions and survival.

## Notes

Supplement 1. **eMethods.** Detailed Description of Statistical Analysis **eTable 1.** Codes to Identify Receipt of Dialysis or Kidney Transplantation Using Administrative Data From Alberta, Canada **eTable 2.** Cohort Formation **eTable 3.** Selected Baseline Patient Characteristics, By Age **eTable 4.** Sex-Specific 5-Year Risks (95% CIs) of Death Overall, Death With or Without Receiving Kidney Replacement Therapy From the Documentation of G5-CKD, Overall and By Baseline Age and Comorbidities, and Both **eTable 5.** Sex-Specific 5-Year Probabilities (95% CIs) of Remaining in G5-CKD, on Dialysis, and Receiving a Kidney Transplant From the Documentation of G5-CKD, Overall and By Baseline Age, Comorbidities, and Both **eTable 6.** Adjusted Hazard Ratios (95% CIs) Comparing Female vs Male for All-Cause Mortality, Transitions From G5-CKD to States of Death, Dialysis, and Transplantation **eFigure 1.** Study Design **eFigure 2.** Sex-Specific All-Cause Mortality Risks Over Time, Overall and By Age
eReferences.

Supplement 2. Data Sharing Statement
