# Supplementary material for: Sex Differences in Mortality and Receipt of Kidney Replacement Therapy Among Adults With Stage 5 Chronic Kidney Disease
Source: JAMA Intern Med. 2025 Nov 17;186(1):89–97. doi: 10.1001/jamainternmed.2025.5979 (PMC12624453; doi:10.1001/jamainternmed.2025.5979)
Supplement: Supplement 1. — eMethods. Detailed Description of Statistical Analysis eTable 1. Codes to Identify Receipt of Dialysis or Kidney Transplantation Using Administrative Data From Alberta, Canada eTable 2. Cohort Formation eTable 3. Selected Baseline Patient Characteristics, By Age eTable 4. Sex-Specific 5-Year Risks (95% CIs) of Death Overall, Death With or Without Receiving Kidney Replacement Therapy From the Documentation of G5-CKD, Overall and By Baseline Age and Comorbidities, and Both eTable 5. Sex-Specific 5-Year Probabilities (95% CIs) of Remaining in G5-CKD, on Dialysis, and Receiving a Kidney Transplant From the Documentation of G5-CKD, Overall and By Baseline Age, Comorbidities, and Both eTable 6. Adjusted Hazard Ratios (95% CIs) Comparing Female vs Male for All-Cause Mortality, Transitions From G5-CKD to States of Death, Dialysis, and Transplantation eFigure 1. Study Design eFigure 2. Sex-Specific All-Cause Mortality Risks Over Time, Overall and By Age eReferences. [file jamainternmed-e255979-s001.pdf]

Supplemental Online Content

Chan C, Sawhney S, Ahmed SB, et al. Sex differences in mortality and receipt of kidney replacement therapy among adults with stage 5 chronic kidney disease. *JAMA Intern Med*. Published online November 17, 2025. doi:10.1001/jamainternmed.2025.5979

eMethods. Detailed Description of Statistical Analysis

eTable 1. Codes to Identify Receipt of Dialysis or Kidney Transplantation Using Administrative Data From Alberta, Canada

eTable 2. Cohort Formation

eTable 3. Selected Baseline Patient Characteristics, By Age

eTable 4. Sex-Specific 5-Year Risks (95% CIs) of Death Overall, Death With or Without Receiving Kidney Replacement Therapy From the Documentation of G5-CKD, Overall and By Baseline Age and Comorbidities, and Both

eTable 5. Sex-Specific 5-Year Probabilities (95% CIs) of Remaining in G5-CKD, on Dialysis, and Receiving a Kidney Transplant From the Documentation of G5-CKD, Overall and By Baseline Age, Comorbidities, and Both

eTable 6. Adjusted Hazard Ratios (95% CIs) Comparing Female vs Male for All-Cause Mortality, Transitions From G5-CKD to States of Death, Dialysis, and Transplantation

eFigure 1. Study Design

eFigure 2. Sex-Specific All-Cause Mortality Risks Over Time, Overall and By Age

eReferences.

This supplementary material has been provided by the authors to give readers additional information about their work.

© 2025 Chan C et al. *JAMA Internal Medicine*.

eMethods. Detailed Description of Statistical Analysis

Estimation of standardized mortality ratios

We used the formula below to calculate standardized mortality ratio (SMR):

$$SMR = \frac{\text{Observed mortality rate in G5} - \text{CKD patients}}{\text{Expected mortality rate in general population}}$$

The mortality rate for G5-CKD patients was calculated by dividing the number of observed deaths in this population by the total person-years at risk. We assumed a Poisson distribution for the number of deaths, and the standard error (SE) of the mortality rate was estimated using the following formula:

$$SE = \frac{\sqrt{Number\ of\ deaths}}{Person - years\ of\ follow - up}$$

$$95\%\ CI\ for\ MR = MR \pm 1.96 * SE$$

To calculate the 95% confidence interval (CI) for the standardized mortality ratio (SMR), we derived the Poisson confidence interval for the observed mortality rate in the G5-CKD cohort and then scaled it by the expected mortality rate from the general population (MR_E). Specifically:

$$95\%\ CI\ for\ SMR = (\frac{Lower\ bound\ of\ MR\_G5CKD}{MR\_E}, \frac{Upper\ bound\ of\ MR\_G5CKD}{MR\_E})$$

Where MR_G5CKD is the mortality rate in G5-CKD patients.

For the general population, the mortality rate was the average annual number of deaths between 2005 and 2021, divided by the average annual population estimates over the same period. Data on deaths in the general population were obtained from Statistics Canada Table 17-10-0006-01 (formerly CANSIM 051-0002),[1] and population estimates were sourced from Table 17-10-0005-01 (formerly CANSIM 051-0001),[2] stratified by sex and 5-year age groups, including age 20 years and older owing to data availability.

Estimation of 95% CI for the female-to-male mortality rate ratio

We reported 95% confidence intervals for the female-to-male mortality rate ratio (MRR) in both the G5-CKD cohort and the general population.

To calculate the standard error (SE) of the log MRR in the G5-CKD cohort, the log of the MRR is approximately normally distributed with SE given by[3]:

$$SE(\ln(MRR)) = \sqrt{\frac{1}{no.of\ deaths\ in\ females} + \frac{1}{no.of\ deaths\ in\ males}}$$

To calculate the 95% confidence interval of MRR:

$$95\%\ CI = \exp\ (\ln(MRR) \pm 1.96 * SE(\ln(MRR)))$$

The same approach was applied to calculate the 95% confidence intervals for the female-to-male mortality rate ratio in the general population.

© 2025 Chan C et al. *JAMA Internal Medicine*.

Estimation of state probabilities
We used the nonparametric Aalen-Johansen method to estimate the sex-specific probabilities of being in a state over time.[4] They included the probabilities of death, receiving dialysis, receiving a kidney transplant, and remaining in the G5-CKD state. The probability of being in the death state was the sum of the probabilities of dying without receiving KRT and dying following KRT initiation. The probability of dying without KRT was estimated by treating KRT initiation and death without KRT as competing events.[4,5] All probabilities were calculated from the time of study entry, including death following KRT initiation. Standard errors for state probabilities were estimated using the forward Greenwood method,[6] and the 95% CIs were constructed using the linear method. Probability differences between males and females were considered statistically significant when their 95% CIs did not overlap.

Adjusted hazard ratios (HRs) comparing female versus male for outcomes
We considered all-cause mortality and three transitions, including G5-CKD to dialysis, G5-CKD to transplantation, G5-CKD to death (i.e., death without receiving KRT), for these analyses. Separate Cox regression models were used to estimate the hazard ratios comparing female versus male for these transitions and all-cause mortality. We followed the recommended procedure to fit transition-specific Cox regression models.[5] Three models were considered. The simple model adjusted for baseline age, diabetes, and cardiovascular disease. The complex model 1 additionally adjusted for dementia, cancer, chronic pulmonary disease, prior hospitalization, outpatient nephrology visit, and severity of albuminuria (categorized normal to mild, moderate, severe, or unmeasured). The complex model 2 included the same covariates as the complex model 1 but excluded individuals with missing albuminuria measurements. There were no other missing data in the outcome variables or covariates included in these models. Note that estimates of transition-specific hazard ratios do not directly correspond to the probability of occupying a given health state. We estimated adjusted HRs in overall cohort and in subgroups defined by age (<45, 45-54, 55-64, 65-74, 75-84, ≥85 years).

We assessed the proportional hazards (PH) assumption for each covariate in all Cox proportional hazards and cause-specific Cox models using both formal Schoenfeld residual tests and graphical methods.[7] For binary or categorical variables with p-values < 0.05 in the PH test, indicating a statistically significant violation, we applied stratified Cox models in accordance with standard survival analysis guidelines.[8] In the cause-specific Cox models assessing the transition from G5-CKD to dialysis among participants aged 75-84 years (three models) and ≥85 years (complex model 2), the PH test for the sex variable yielded p-values < 0.05. Visual inspection of log-minus-log survival curves and scaled Schoenfeld residual plots for this variable supported the PH assumption.

Estimation of survival time, median time in a state, and median follow-up time
We used the Kaplan-Meier method to estimate all-cause mortality risk and median survival time, treating all-cause mortality as the event of interest.[9] The same method was applied to estimate the median time spent in the non-KRT G5-CKD state, considering transitions to dialysis, transplantation, or death as events. Among individuals who initiated dialysis, we estimated the median time spent in the dialysis state, with transitions to transplantation or death treated as events. Among transplant recipients during follow-up, we did not estimate their median time spent alive post-transplant due to relatively small number of deaths. Median follow-up time was estimated using the reverse Kaplan-Meier method, treating censoring as the event of interest.[10]

eTable 1. Codes to Identify Receipt of Dialysis or Kidney Transplantation Using Administrative Data From Alberta, Canada

a) Physician claims: Alberta Health Care Insurance Plan, Medical Procedure codes

Codes	Code description
Dialysis	
13.99A	Hemodialysis treatment, unstable patient
13.99B	Hemodialysis treatment, stable patient
13.99C	Assessment and management of an unstable patient with chronic renal failure treated by peritoneal dialysis
13.99D	Assessment and management of a stable patient with chronic renal failure treated by peritoneal dialysis
13.99O	Management of dialysis patients on home dialysis or receiving treatment in a remote hemodialysis unit (per week)
13.99OA	Management of patient on hemodialysis or peritoneal dialysis (per week)
13.99AB	Dialysis therapy, any modality, in the intensive care unit
Transplantation	
67.5	Transplant of kidney
67.59	Other kidney transplantation
67.59A	Renal transplantation (homo, hetero, auto)

b) Discharge Abstract Database: Canadian Classification of Health Intervention and ICD-9-CM procedure codes

Codes	Code description
For transplantation: Canadian Classification of Health Intervention codes	
1.PC.85.	Transplant, kidney
1.PC.85.LA-XX-J	Using living donor (allogenic or syngeneic) kidney
1.PC.85.LA-XX-K	Using decreased donor kidney
1.OK.85.XU-XX-K	Transplant, pancreas with duodenum and kidney with exocrine drainage via bladder
1.OK.85.XV-XX-K	Transplant, pancreas with duodenum and kidney with exocrine drainage via intestine with homograft
For transplantation: ICD-9-CM procedure codes	
55.69	Other kidney transplantation

Note: Maintenance dialysis recipients were identified using data from either the provincial kidney care program or physician claims databases. The kidney care program database included episodes of maintenance dialysis lasting more than 90 days and those initiating dialysis for chronic kidney failure who died or received a kidney transplant before 90 days. Episodes of acute dialysis were excluded.

For the physician claims database, maintenance dialysis was defined by a continuous period of dialysis claims lasting more than 90 days, with no gap between claims exceeding 90 days. For 13.99C or 13.99D claims, the permissible gap was relaxed to no more than 182 days. If there was a continuous period of dialysis claims for less than 90 days followed by a kidney transplant, the date of dialysis initiation was considered as the date of kidney replacement for kidney failure.

© 2025 Chan C et al. *JAMA Internal Medicine*.

eTable 2. Cohort Formation

	All	Female	Male	Other[a]
Number of residents registered under the Alberta Health Care Insurance Plan April 1, 1994 – March 31, 2021	5,434,195	2,667,272 (49.1)	2,766,913 (50.9)	10
**Exclusion criteria (N excluded)**:	*5,426,689*	*2,663,887 (49.1)*	*2,762,792 (50.9)*	*10*
No serum creatinine measurements (available May 1, 2002 – March 31, 2021)	1,550,936	655,083 (42.2)	895,852 (57.8)	5
No outpatient serum creatinine measurements	162,565	57,965 (35.7)	104,600 (64.3)	5
Never had an outpatient eGFR measurement <15 mL/min/1.73 m^2	3,685,395	1,937,132 (52.6)	1,748,254 (47.4)	9
Only 1 outpatient eGFR <15 mL/min/1.73 m^2	11,194	6,058 (54.1)	5,136 (45.9)	5
Did not meet the sustained eGFR criterion for G5-CKD	8327	4,234 (50.85)	4,093 (49.15)	5
Age <18 years on index date	16	10 (62.5)	6 (37.5)	5
Index date was not between April 1, 2005, and March 31, 2019	4855	2,116 (43.6)	2,739 (56.4)	5
Initiated maintenance dialysis or received a kidney transplant on or prior to index date	3370	1,275 (37.8)	2,095 (62.2)	5
Exited (death, outmigration) on or before index date	31	14 (45.2)	17 (54.8)	5
**Cohort size (N)**	**7506[b]**	**3385 (45.1)**	**4121 (54.9)**	**0**

Values for female and male columns are n (%).
[a]There were ten people whose sex marker was "other" in the 2021 Provincial Registry database and their eGFR measurements were calculated using male and female sex, respectively. Numbers in each step of the exclusion sequence are rounded to nearest 5 to prevent reporting numbers less than 5.

Among individuals who did not meet the study eligibility criteria, female residents were more likely than males to have serum creatinine testing in the outpatient setting but were less likely to have an outpatient eGFR <15 mL/min/1.73 m^2 or a history of maintenance KRT.

Among 7506 individuals with incident G5-CKD, five (<0.1%) had changed their legal sex marker before cohort entry.

[b]There were 65 (0.9%) individuals who out-migrated from the province during the follow-up period.

© 2025 Chan C et al. *JAMA Internal Medicine*.

eTable 3. Selected Baseline Patient Characteristics, By Age

Characteristic	Age 18-64 y (N=2745)		Age 65-84 y (N=3555)		Age ≥85 y (N=1206)	
	**Male**	**Female**	**Male**	**Female**	**Male**	**Female**
N	1620	1125	1973	1582	528	678
Days of qualifying period, median (IQR)	105 (96-120)	106 (96-124)	105 (96-121)	108 (97-127)	112 (97-133)	119 (101-168)
Albuminuria[a]						
A1	51 (3.3)	56 (5.0)	202 (6.5)	217 (13.7)	125 (13.4)	156 (23.0)
A2	184 (10.2)	174 (15.5)	384 (17.4)	359 (22.7)	160 (26.1)	162 (23.9)
A3	1188 (80.7)	846 (75.2)	1252 (71.1)	908 (57.4)	292 (51.1)	275 (40.6)
Unmeasured	94 (5.8)	49 (4.4)	99 (5.0)	98 (6.2)	49 (9.3)	85 (12.5)
Diabetes	877 (54.1)	605 (53.8)	1318 (66.8)	1051 (66.4)	227 (43.0)	307 (45.3)
CVD						
Myocardial infarction	115 (7.1)	60 (5.3)	311 (15.8)	215 (13.6)	90 (17.0)	93 (13.7)
Heart failure	362 (22.3)	268 (23.8)	809 (41.0)	763 (48.2)	289 (54.7)	399 (58.8)
Stroke or TIA	117 (7.2)	75 (6.7)	287 (14.5)	220 (13.9)	90 (17.0)	117 (17.3)
PVD	73 (4.5)	58 (5.2)	209 (10.6)	141 (8.9)	55 (10.4)	50 (7.4)
Diabetes and CVD						
Neither	632 (39.0)	447 (39.7)	389 (19.7)	276 (17.4)	114 (21.6)	125 (18.4)
Diabetes only	478 (29.5)	328 (29.2)	500 (25.3)	370 (23.4)	69 (13.1)	89 (13.1)
CVD only	111 (6.9)	73 (6.5)	266 (13.5)	255 (16.1)	187 (35.4)	246 (36.3)
Both	399 (24.6)	277 (24.6)	818 (41.5)	681 (43.0)	158 (29.9)	218 (32.2)
Chronic pulmonary disease	261 (16.1)	234 (20.8)	704 (35.7)	552 (34.9)	226 (42.8)	217 (32.0)
Cancer	213 (13.1)	176 (15.6)	570 (28.9)	332 (21.0)	162 (30.7)	123 (18.1)
Dementia	32 (2.0)	23 (2.0)	172 (8.7)	204 (12.9)	119 (22.5)	234 (34.5)
Outpatient nephrology visit	1486 (91.7)	1025 (91.1)	1682 (85.3)	1228 (77.6)	312 (59.1)	308 (45.4)
Hospitalization	578 (35.7)	417 (37.1)	809 (41.0)	751 (47.5)	261 (49.4)	306 (45.1)

Values are n (%) otherwise specified. CVD, cardiovascular disease; eGFR, estimated glomerular filtration rate (mL/min/1.73 m^2); IQR, interquartile range; SD, standard deviation, TIA, transient ischemic attack; PVD, peripheral vascular disease.

[a] Albuminuria severity was categorized as normal to mild (A1), moderate (A2), severe (A3), or unmeasured, corresponding to albumin-to-creatinine ratio (ACR) values of <30, 30-300, or >300 mg/g; protein-to-creatinine ratio (PCR) values of <150, 150-500, or >500 mg/g; and urine dipstick protein levels of negative/trace, 1+, or ≥2+. If multiple albuminuria measurement methods were available, ACR was used preferentially, followed by PCR and urine dipstick. Multiply by 0.113 to convert mg/g to mg/mmol.

© 2025 Chan C et al. *JAMA Internal Medicine*.

eTable 4. Sex-Specific 5-Year Risks (95% CIs) of Death Overall, Death With or Without Receiving Kidney Replacement Therapy From the Documentation of G5-CKD, Overall and By Baseline Age and Comorbidities, and Both

	**Death Overall**		**Death without KRT**		**Death following KRT**	
	Male	Female	Male	Female	Male	Female
**All**	**51.9 (50.3-53.5)**	**58.9 (57.2-60.7)**	**28.3 (26.9-29.7)**	**39.9 (38.3-41.6)**	**23.6 (22.8-24.4)**	**19.0 (18.5-19.4)**
**By age, y**						
18-44	8.2 (5.2-11.2)	14.6 (9.9-19.4)	1.1 (0-2.2)	0.4 (0-1.2)	7.1 (4.3-9.8)	14.3 (9.6-18.9)
45-54	19.9 (16.0-23.8)	25.7 (20.4-31.0)	5.0 (2.9-7.1)	7.6 (4.5-10.7)	14.9 (11.6-18.2)	18.1 (13.8-22.4)
55-64	35.9 (32.3-39.5)	35.0 (30.7-39.2)	9.0 (6.9-11.0)	9.0 (6.6-11.5)	26.9 (24.0-29.9)	25.9 (22.5-29.4)
65-74	53.8 (50.4-57.2)	57.8 (53.8-61.8)	**18.7 (16.2-21.2)**	**27.3 (23.8-30.8)**	**35.1 (32.8-37.4)**	**30.5 (28.6-32.5)**
75-84	71.7 (68.8-74.7)	74.0 (71.1-77.0)	**44.8 (41.7-48.0)**	**55.7 (52.5-59.0)**	**26.9 (25.9-28.0)**	**18.3 (16.9-19.8)**
≥85	91.5 (89.0-94.0)	90.1 (87.7-92.5)	81.1 (77.7-84.6)	85.7 (82.9-88.5)	**10.4 (8.0-12.7)**	**4.4 (2.9-5.8)**
**By comorbidities**						
DM-/CVD-	32.7 (29.8-35.5)	33.4 (30.1-36.7)	19.2 (16.9-21.6)	23.7 (20.8-26.7)	**13.4 (11.8-15.1)**	**9.6 (8.1-11.2)**
DM+/CVD-	42.8 (39.6-46.1)	47.7 (44.0-51.5)	**18.9 (16.5-21.4)**	**27.7 (24.4-31.0)**	23.9 (21.8-26.1)	20.0 (18.2-21.9)
DM-/CVD+	**68.5 (64.5-72.6)**	**76.9 (73.3-80.5)**	**47.7 (43.5-52.0)**	**63.2 (59.1-67.2)**	**20.8 (19.7-21.9)**	**13.8 (11.9-15.6)**
DM+/CVD+	**67.9 (65.2-70.6)**	**75.6 (72.9-78.2)**	**34.9 (32.3-37.4)**	**48.1 (45.2-51.0)**	**33.1 (32.5-33.7)**	**27.4 (26.2-28.7)**

DM, diabetes; CVD, cardiovascular disease; (-) indicates absence of comorbidity; (+) indicates presence of comorbidity.
Bolded values indicate statistically significant differences in probability between males and females.

© 2025 Chan C et al. *JAMA Internal Medicine*.

eTable 4. Sex-Specific 5-Year Risks (95% CIs) of Death Overall, Death With or Without Receiving Kidney Replacement Therapy From the Documentation of G5-CKD, Overall and By Baseline Age and Comorbidities, and Both (continued)

	**Death Overall**		**Death without KRT**		**Death following KRT**	
	Male	Female	Male	Female	Male	Female
**By age and comorbidities**						
**Age 18-64 y**						
DM-/CVD-	9.6 (7.1-12.0)	10.5 (7.5-13.5)	3.4 (1.9-4.9)	3.8 (2.0-5.7)	6.2 (4.2-8.1)	6.7 (4.3-9.1)
DM+/CVD-	26.1 (21.8-30.4)	29.1 (23.7-34.5)	4.3 (2.4-6.2)	8.5 (5.3-11.7)	21.8 (18.0-25.6)	20.6 (16.3-24.9)
DM-/CVD+	28.5 (19.3-37.6)	38.9 (27.0-50.8)	10.2 (4.3-16.2)	10.2 (2.5-17.8)	18.2 (11.3-25.2)	28.7 (19.7-37.8)
DM+/CVD+	47.2 (41.9-52.5)	51.8 (45.3-58.3)	11.2 (8.0-14.5)	8.4 (5.0-11.7)	36.1 (31.9-40.2)	43.4 (37.8-49.0)
**Age 65-74 y**						
DM-/CVD-	42.8 (35.5-50.1)	36.0 (26.8-45.3)	13.6 (8.6-18.5)	18.6 (11.1-26.2)	**29.2 (23.8-34.6)**	**17.4 (12.0-22.9)**
DM+/CVD-	41.7 (35.4-48.0)	45.4 (37.6-53.2)	14.0 (9.7-18.3)	20.3 (14.1-26.6)	27.3 (22.7-31.9)	25.1 (20.4-29.8)
DM-/CVD+	53.7 (43.3-64.1)	65.4 (53.3-77.6)	26.7 (17.6-35.7)	38.5 (25.6-51.3)	27.1 (21.9-32.2)	27.0 (22.8-31.2)
DM+/CVD+	68.3 (63.1-73.5)	72.3 (66.8-77.7)	**22.6 (18.3-26.9)**	**32.9 (27.3-38.4)**	**45.9 (43.0-48.9)**	**39.5 (38.4-40.5)**
**Age 75-84 y**						
DM-/CVD-	63.1 (55.8-70.3)	54.8 (46.7-62.9)	40.0 (32.7-47.2)	38.7 (30.8-46.6)	**23.1 (22.1-24.1)**	**16.3 (14.5-18.2)**
DM+/CVD-	67.4 (60.5-74.4)	63.9 (56.5-71.2)	38.8 (32.1-45.6)	45.4 (37.7-53.1)	**28.6 (27.0-30.2)**	**18.5 (16.4-20.6)**
DM-/CVD+	75.0 (67.9-82.0)	76.1 (70.0-82.3)	46.8 (38.9-54.8)	60.2 (53.1-67.3)	**28.1 (24.5-31.8)**	**15.9 (12.4-19.4)**
DM+/CVD+	76.8 (72.6-81.0)	85.4 (81.6-89,1)	**49.5 (44.5-54.4)**	**64.8 (59.9-69.7)**	**27.4 (24.8-29.9)**	**20.5 (17.4-23.6)**
**Age ≥85 y**						
DM-/CVD-	86.8 (80.5-93.2)	83.8 (77.2-90.4)	78.1 (70.1-86.1)	79.8 (72.3-87.2)	8.8 (3.9-13.7)	4.0 (0.6-7.4)
DM+/CVD-	85.1 (76.2-94.0)	87.7 (80.1-95.3)	75.7 (64.5-86.8)	78.7 (68.9-88.4)	8.9 (2.1-15.6)	9.0 (2.9-15.1)
DM-/CVD+	93.1 (89.3-96.8)	91.8 (88.1-95.5)	81.4 (75.6-87.2)	87.7 (83.3-92.2)	**11.7 (7.2-16.1)**	**4.1 (1.6-6.6)**
DM+/CVD+	95.8 (92.2-99.3)	92.5 (88.8-96.2)	85.2 (79.1-91.2)	89.7 (85.4-94.1)	**10.6 (5.7-15.5)**	**2.8 (0.5-5.1)**

DM, diabetes; CVD, cardiovascular disease; (-) indicates absence of comorbidity; (+) indicates presence of comorbidity.
Bolded values indicate statistically significant differences in probability between males and females.

© 2025 Chan C et al. *JAMA Internal Medicine*.

eTable 5. Sex-Specific 5-Year Probabilities (95% CIs) of Remaining in G5-CKD, on Dialysis, and Receiving a Kidney Transplant From the Documentation of G5-CKD, Overall and By Baseline Age, Comorbidities, and Both

	**G5-CKD**		**Dialysis**		**Transplantation**	
	Male	Female	Male	Female	Male	Female
**All**	**5.7 (5.0-6.5)**	**9.3 (8.2-10.3)**	**27.8 (26.3-29.3)**	**23.3 (21.8-24.8)**	**14.5 (13.4-15.7)**	**8.5 (7.5-9.5)**
**By age, y**						
18-44	3.4 (1.4-5.4)	4.5 (1.6-7.3)	32.7 (27.4-37.9)	38.9 (32.3-45.6)	**55.7 (50.3-61.2)**	**42.0 (35.4-48.5)**
45-54	4.4 (2.3-6.5)	5.1 (2.5-7.8)	41.7 (36.7-46.6)	42.7 (36.5-48.8)	34.0 (29.4-38.7)	26.5 (21.2-31.9)
55-64	3.8 (2.4-5.3)	7.4 (5.1-9.7)	38.3 (34.6-42.0)	42.4 (37.9-46.9)	**22.0 (18.9-25.0)**	**15.2 (12.0-18.4)**
65-74	8.3 (6.4-10.2)	11.1 (8.5-13.7)	31.0 (27.8-34.3)	28.4 (24.7-32.1)	**6.9 (5.2-8.6)**	**2.6 (1.3-4.0)**
75-84	**7.1 (5.4-8.8)**	**13.1 (10.8-15.4)**	**21.2 (18.5-23.9)**	**12.8 (10.5-15.0)**	0	0.1 (0-0.3)
≥85	4.3 (2.5-6.2)	7.3 (5.2-9.4)	4.2 (2.4-6.0)	2.6 (1.4-3.9)	0	0
**By comorbidities**						
DM-/CVD-	**6.9 (5.3-8.5)**	**13.3 (10.9-15.8)**	30.3 (27.4-33.2)	31.3 (28.0-34.7)	**30.2 (27.3-33.0)**	**21.9 (19.0-24.9)**
DM+/CVD-	**5.3 (3.8-6.8)**	**10.0 (7.7-12.3)**	35.9 (32.7-39.1)	33.7 (30.1-37.3)	**15.9 (13.6-18.3)**	**8.6 (6.5-10.7)**
DM-/CVD+	8.1 (5.8-10.5)	10.1 (7.6-12.7)	**18.9 (15.4-22.4)**	**10.1 (7.6-12.7)**	4.5 (2.7-6.3)	2.8 (1.4-4.2)
DM+/CVD+	4.1 (2.9-5.2)	5.5 (4.1-6.9)	**23.4 (21.0-25.8)**	**17.3 (15.0-19.7)**	**4.7 (3.4-5.9)**	**1.6 (0.8-2.3)**

DM, diabetes; CVD, cardiovascular disease; (-) indicates absence of comorbidity; (+) indicates presence of comorbidity.
Bolded values indicate statistically significant differences in probability between males and females.

© 2025 Chan C et al. *JAMA Internal Medicine*.

eTable 5. Sex-Specific 5-Year Probabilities (95% CIs) of Remaining in G5-CKD, on Dialysis, and Receiving a Kidney Transplant From the Documentation of G5-CKD, Overall and By Baseline Age, Comorbidities, and Both (continued)

	**G5-CKD**		**Dialysis**		**Transplantation**	
	Male	Female	Male	Female	Male	Female
**By age and comorbidities**						
**Age 18-64 y**						
DM-/CVD-	4.2 (2.5-6.0)	7.8 (5.1-10.5)	35.8 (31.7-39.8)	41.8 (36.9-46.8)	**50.4 (46.3-54.6)**	**39.9 (35.0-44.7)**
DM+/CVD-	3.9 (2.1-5.8)	4.5 (2.0-7.0)	41.1 (36.2-46.0)	47.7 (41.7-53.7)	**28.9 (24.5-33.3)**	**18.7 (14.1-23.3)**
DM-/CVD+	9.7 (4.0-15.5)	8.1 (1.6-14.7)	42.0 (31.7-52.2)	31.5 (19.9-43.2)	19.8 (11.7-28.0)	21.4 (11.4-31.5)
DM+/CVD+	1.5 (0.1-2.8)	4.8 (2.2-7.4)	36.9 (31.7-42.1)	37.1 (30.7-43.4)	14.4 (10.7-18.1)	6.4 (3.3-9.4)
**Age 65-74 y**						
DM-/CVD-	11.4 (6.6-16.1)	22.1 (14.0-30.1)	32.0 (25.0-39.1)	34.9 (25.6-44.2)	13.8 (8.7-18.9)	7.0 (2.0-12.0)
DM+/CVD-	8.4 (4.8-12.0)	13.6 (8.2-18.9)	39.8 (33.5-46.1)	37.3 (29.6-44.9)	10.1 (6.4-13.9)	3.8 (0.8-6.8)
DM-/CVD+	11.1 (4.7-17.5)	5.8 (0-12.1)	31.0 (21.3-40.8)	27.0 (15.6-38.5)	4.2 (0.2-8.1)	1.7 (0-4.9)
DM+/CVD+	6.0 (3.4-8.6)	6.4 (3.3-9.4)	23.9 (19.1-28.8)	20.9 (16.0-25.9)	1.7 (0.2-3.2)	0.4 (0-1.2)
**Age 75-84 y**						
DM-/CVD-	**10.9 (6.2-15.6)**	**24.4 (17.5-31.4)**	26.0 (19.4-32.7)	20.2 (13.6-26.7)	0	0.7 (0-2.0)
DM+/CVD-	**3.7 (0.9-6.4)**	**17.2 (11.2-23.1)**	28.9 (22.2-35.6)	19.0 (13.0-24.9)	0	0
DM-/CVD+	11.9 (6.7-17.1)	16.9 (11.5-22.2)	13.2 (7.6-18.8)	7.0 (3.3-10.8)	0	0
DM+/CVD+	5.3 (2.9-7.6)	5.1 (2.8-7.3)	**17.9 (14.1-21.7)**	**9.6 (6.4-12.7)**	0	0
**Age ≥85 y**						
DM-/CVD-	7.0 (2.2-11.9)	10.5 (5.0-16.1)	6.1 (1.7-10.5)	5.7 (1.6-9.8)	0	0
DM+/CVD-	8.3 (1.3-15.3)	8.4 (1.7-15.0)	6.6 (0.5-12.6)	3.9 (0-8.4)	0	0
DM-/CVD+	2.9 (0.4-5.4)	6.2 (2.9-9.5)	**4.0 (1.1-6.9)**	**2.0 (0.3-3.8)**	0	0
DM+/CVD+	2.7 (0-5.6)	6.1 (2.7-9.6)	1.5 (0-3.6)	1.4 (0-2.9)	0	0

DM, diabetes; CVD, cardiovascular disease; (-) indicates absence of comorbidity; (+) indicates presence of comorbidity.
Bolded values indicate statistically significant differences in probability between males and females.

© 2025 Chan C et al. *JAMA Internal Medicine*.

eTable 6. Adjusted Hazard Ratios (95% CIs) Comparing Female vs Male for All-Cause Mortality, Transitions From G5-CKD to States of Death, Dialysis, and Transplantation

Outcomes	Age (years)	Simple model	Complex model 1	Complex model 2
All-cause mortality	All	0.94 (0.73-1.21)	1.01 (0.59-1.73)	0.94 (0.53-1.64)
	18-44	**1.86 (1.23-2.80)**	**1.84 (1.21-2.80)**	**1.69 (1.10-2.59)**
	45-54	**1.34 (1.03-1.73)**	1.23 (0.95-1.61)	1.18 (0.90-1.56)
	55-64	1.04 (0.89-1.21)	1.04 (0.89-1.22)	1.03 (0.88-1.22)
	65-74	1.06 (0.94-1.19)	1.04 (0.92-1.18)	1.05 (0.93-1.19)
	75-84	1.03 (0.70-1.51)	0.72 (0.35-1.51)	0.72 (0.35-1.50)
	≥85	0.91 (0.81-1.03)	**0.87 (0.77-0.98)**	0.81 (0.33-1.98)
Transitions from G5-CKD to:				
Death	All	1.03 (0.72-1.46)	0.93 (0.86-1.01)	0.94 (0.86-1.02)
	18-44	0.68 (0.13-3.43)	0.35 (0.05-2.67)	0.06 (0.00-1.56)
	45-54	1.31 (0.73-2.35)	1.16 (0.61-2.20)	0.97 (0.49-1.91)
	55-64	0.79 (0.55-1.13)	0.79 (0.55-1.15)	0.81 (0.55-1.18)
	65-74	**1.23 (1.00-1.50)**	1.20 (0.98-1.47)	1.21 (0.98-1.49)
	75-84	0.94 (0.83-1.07)	0.95 (0.83-1.08)	0.95 (0.83-1.09)
	≥85	0.91 (0.80-1.03)	0.88 (0.77-1.00)	0.95 (0.83-1.09)
Dialysis	All	**0.73 (0.68-0.78)**	**0.80 (0.75-0.85)**	**0.80 (0.75-0.85)**
	18-44	0.95 (0.80-1.13)	0.98 (0.82-1.18)	0.96 (0.80-1.16)
	45-54	0.87 (0.74-1.02)	0.90 (0.76-1.06)	0.91 (0.77-1.08)
	55-64	**0.80 (0.71-0.91)**	**0.86 (0.76-0.97)**	**0.84 (0.74-0.96)**
	65-74	**0.74 (0.66-0.84)**	**0.80 (0.70-0.90)**	**0.80 (0.71-0.91)**
	75-84	**0.58 (0.50-0.67)**	**0.68 (0.59-0.80)**	**0.70 (0.60-0.82)**
	≥85	**0.48 (0.34-0.69)**	**0.60 (0.41-0.87)**	**0.67 (0.57-0.78)**
Transplantation	All	**0.56 (0.42-0.76)**	**0.58 (0.43-0.78)**	**0.57 (0.42-0.76)**

The simple Cox model adjusted for baseline age (continuous), diabetes, and cardiovascular disease.
The complex model 1 additionally adjusted for cancer, dementia, chronic pulmonary disease, hospitalization, outpatient nephrology visit, and albuminuria (categorized normal to mild, moderate, severe, or unmeasured).
The complex model 2 included the same covariates as the complex model 1 but excluded individuals with missing albuminuria measurements.
Bolded values indicate statistically significant differences in hazards between males and females.

© 2025 Chan C et al. *JAMA Internal Medicine*.

eFigure 1. Study Design

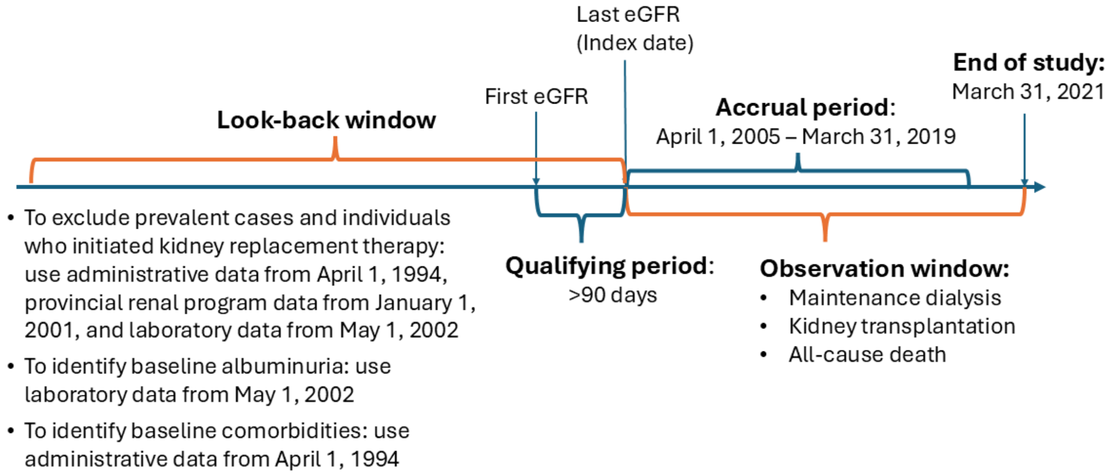

© 2025 Chan C et al. *JAMA Internal Medicine*.

eFigure 2. Sex-Specific All-Cause Mortality Risks Over Time, Overall and By Age

Panel A. Overall

G5-CKD cohort (N=7506)

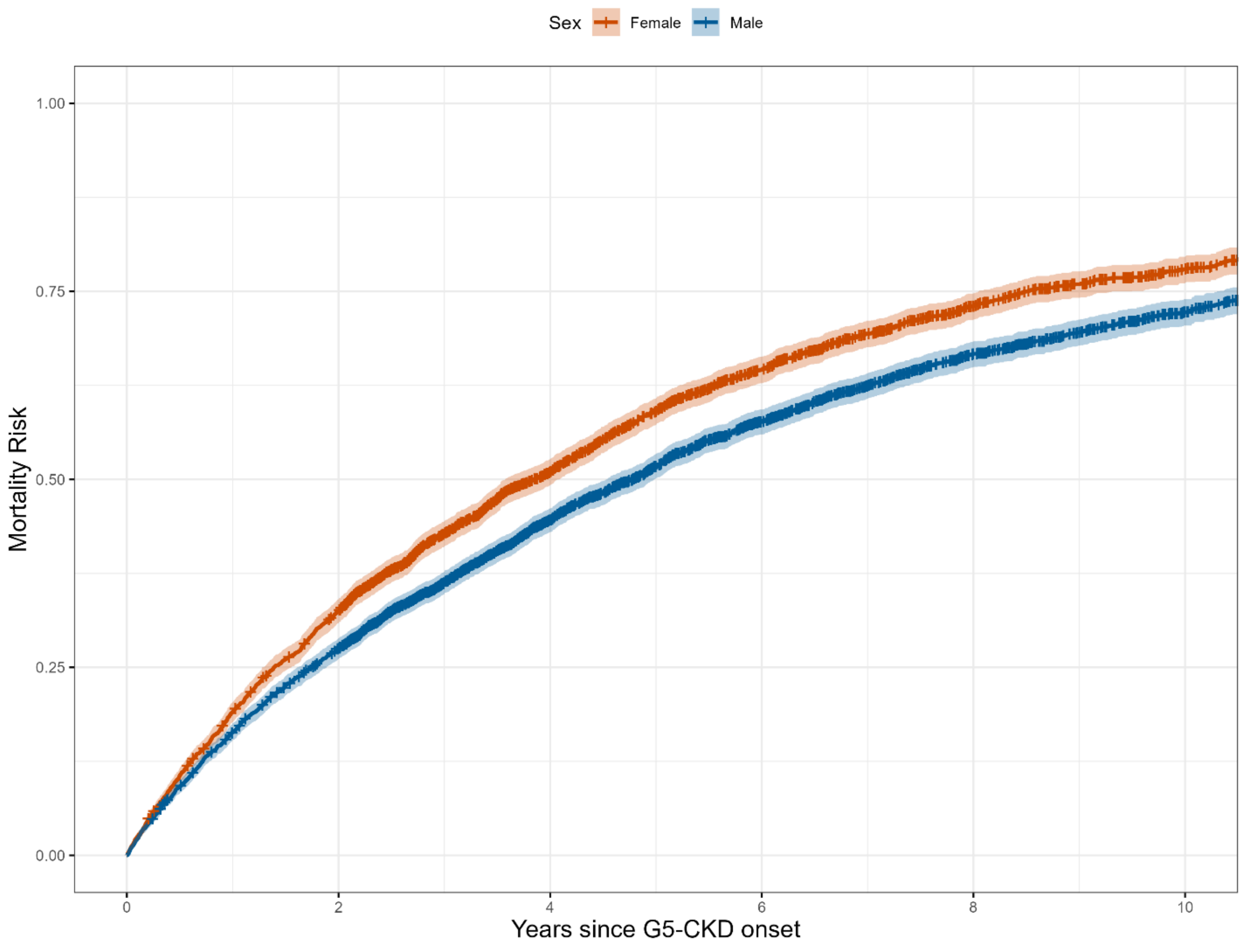

© 2025 Chan C et al. *JAMA Internal Medicine*.

Panel B. By age

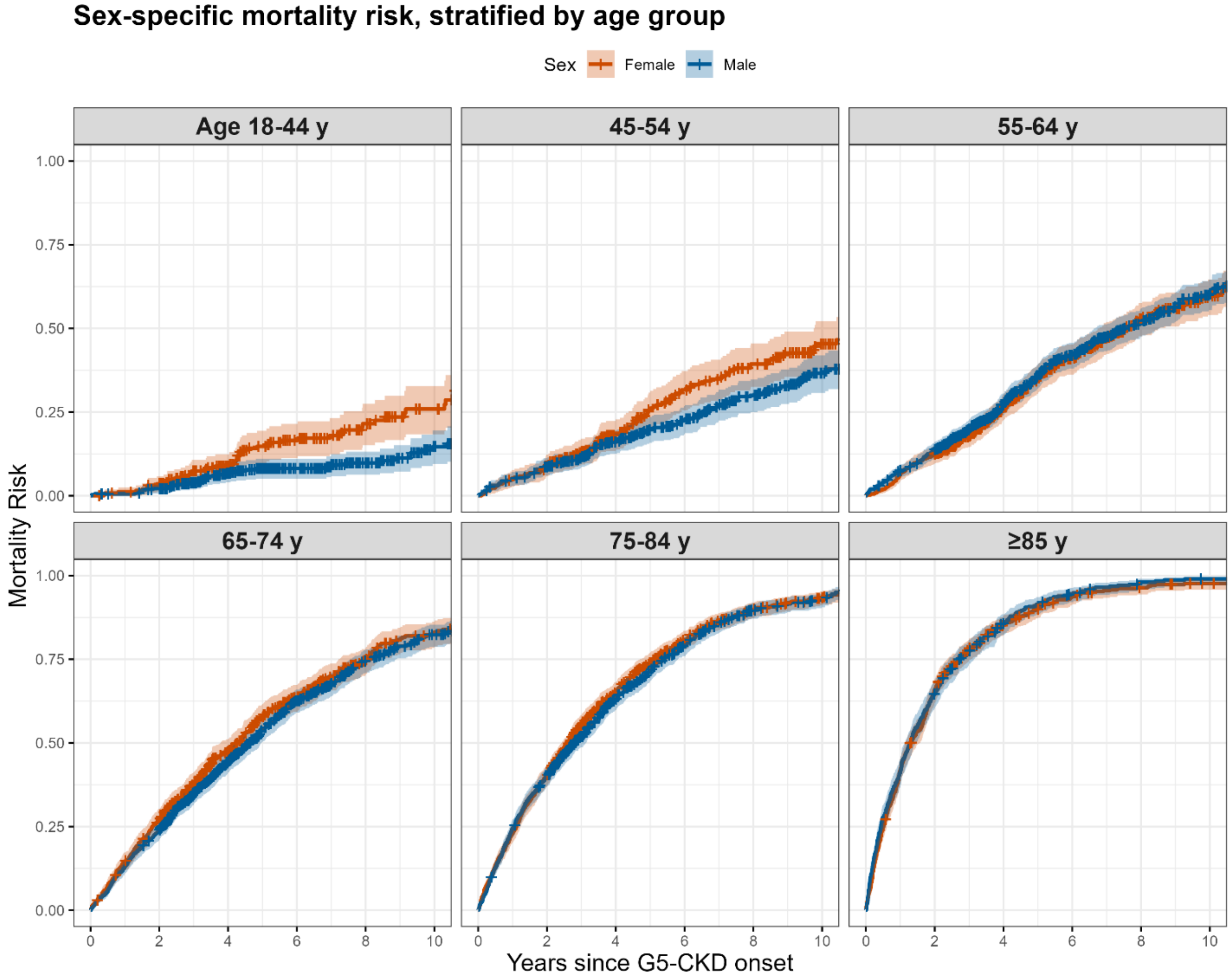

Legend: Mortality risks were estimated using the Kaplan-Meier method. Shaded areas represent 95% confidence intervals.

© 2025 Chan C et al. *JAMA Internal Medicine*.

eReferences.

1. Statistics Canada. Table 17-10-0006-01 Estimates of deaths, by age and gender, annual. https://doi.org/10.25318/1710000601-eng. Accessed June 27, 2025.
2. Statistics Canada. Table 17-10-0005-01 Population estimates on July 1, by age and gender. https://doi.org/10.25318/1710000501-eng. Accessed June 27, 2025.
3. Rothman KJ. Epidemiology: An introduction. 2nd ed. New York: Oxford University Press, 2012.
4. Aalen OO, Johansen S. An Empirical Transition Matrix for Non-Homogeneous Markov Chains Based on Censored Observations. Scandinavian Journal of Statistics. 1978;5(3):141-150.
5. Putter H, Fiocco M, Geskus RB. Tutorial in biostatistics: competing risks and multi-state models. *Stat Med.* 2007;26(11):2389-430.
6. de Wreede LC, Fiocco M, Putter H. The mstate package for estimation and prediction in non- and semi-parametric multi-state and competing risks models. *Comput Methods Programs Biomed.* 2010;99(3):261-74.
7. Hess KR. Graphical methods for assessing violations of the proportional hazards assumption in Cox regression. *Stat Med.* 1995;14(15):1707-23.
8. Therneau TM, Grambsch PM. Modeling survival data: extending the Cox model. New York: Springer, 2000.
9. Kaplan EL, Meier P. Nonparametric Estimation from Incomplete Observations. Source: Journal of the American Statistical Association. 1958;53(282):457-481.
10. Schemper M, Smith TL. A note on quantifying follow-up in studies of failure time. *Control Clin Trials.* 1996;17(4):343-6.

© 2025 Chan C et al. *JAMA Internal Medicine*.
