# Supplementary material for: Sex Differences in Mortality and Receipt of Kidney Replacement Therapy Among Adults With Stage 5 Chronic Kidney Disease
Source: JAMA Intern Med. 2025 Nov 17;186(1):89–97. doi: 10.1001/jamainternmed.2025.5979 (PMC12624453; doi:10.1001/jamainternmed.2025.5979)
Supplement: Supplement 2. — Data Sharing Statement [file jamainternmed-e255979-s002.pdf]

Data Sharing Statement

Chan. Sex Differences in Mortality and Receipt of Kidney Replacement Therapy Among Adults With Stage 5 Chronic Kidney Disease. *JAMA Intern Med*. Published November 17, 2025. doi:10.1001/jamainternmed.2025.5979

Data
Data available: No
